# Concurrent atypical diffused tuberculosis and macrorhabdosis in a canary (*Serinus canaria*)

**Published:** 2015-03-15

**Authors:** Seyed Ahmad Madani, Mohammad Reza Haddad-Marandi, Fatemeh Arabkhazaeli

**Affiliations:** 1 *Department of Avian Diseases, Faculty of Veterinary Medicine, University of Tehran, Tehran, Iran; *; 2 *Avian Diseases Research Center, School of Veterinary Medicine, Shiraz University, Shiraz, Iran;*; 3 *Department of Parasitology, Faculty of Veterinary Medicine, University of Tehran, Tehran, Iran.*

**Keywords:** Atypical tuberculosis, Canary, Macrorhabdosis, Megabacteriosis, Mycobacteriosis

## Abstract

A dead canary from a mixed species zoological garden was presented for diagnostic necropsy. Cachexia with prominent atrophy of pectoral muscles, yellowish brown discoloration of the liver and kidney, dark brown to black intestinal contents and moderate proventricular dilatation with some degree of catarrhal gastritis were the significant macroscopic findings. Parenchymatous organs like the liver, the spleen, the lung and the kidneys were extremely affected by massive diffuse necrosis and heavy infiltration of mononuclear inflammatory cells, histopathologically. Many giant bacilli resembling *Macrorrhabdus ornithogaster* were seen microscopically in the wet smear of the isthmus mucosa. Ghost-like unstained bacilli were revealed in the Giemsa stained contact smears of the liver and spleen. No typical mycobacterial granulomatous lesion was found in different tissues, but in Ziehl-Neelsen stained thin layer histologic sections from the liver, spleen, lung and kidney, numerous acid fast organisms were diffusely distributed. The case was diagnosed an atypical avian tuberculosis with concurrent macrorhabdosis. *Mycobacterium *sp. are capable of giving rise to a progressive disease in humans, especially in immunocompromised individuals. Cases of avian tuberculosis might be overlooked for lack of pathognomonic lesions suggestive of mycobacteriosis.

## Introduction

Avian tuberculosis is a contagious chronic bacterial disease;^1^ most often caused by *Mycobacterium avium-intracellulare* known as *M. avium *complex^2^ and *M. genavense*.^2^^-^^6^ However, several mycobacterial species, such as *M. avium* ^7^^-^^9^ *M. tuberculosis*,^10^^,^^11^ *M. Bovis, M. fortuitum*, *M. gordonae *and *M. nonchromogenicum*^2^ have been isolated from exotic and pet birds. Different species of *Mycobacterium* are responsible for various progressive diseases in human especially in immunocompromised hosts and are refractory to treatment.^12^ There are no pathogonomic clinical signs for avian mycobacteriosis.^1^ Avian tuberculosis has been divided into three types regarding the gross lesions: classical form with tubercles in many organs*,* paratuberculosis form with typical lesions in the intestinal tract and non-tuberculous form known as diffused non-tuberculous mycobacteriosis or atypical mycobacteriosis.^13^^,^^14^ Demonstration of acid-fast bacilli in affected organs using Ziehl-Neelsen staining, isolation of the *Mycobacterium* sp., serological tests and molecular techniques have been used to confirm the diagnosis.^12^ Due to lack of specific postmortem finding and the fastidious growing of some mycobacterial species or non-culturable mycobacteria,^4^^,^^8^^,^^15^^,^^16^ the causative agents of avian mycobacteriosis in pet birds are rarely identified.^2^^,^^6^

In this article, we presented a case of canary atypical tuberculosis with concurrent mycotic gastritis due to *M. ornithogaster *infection.

## Case Description

**Fig. 1:**
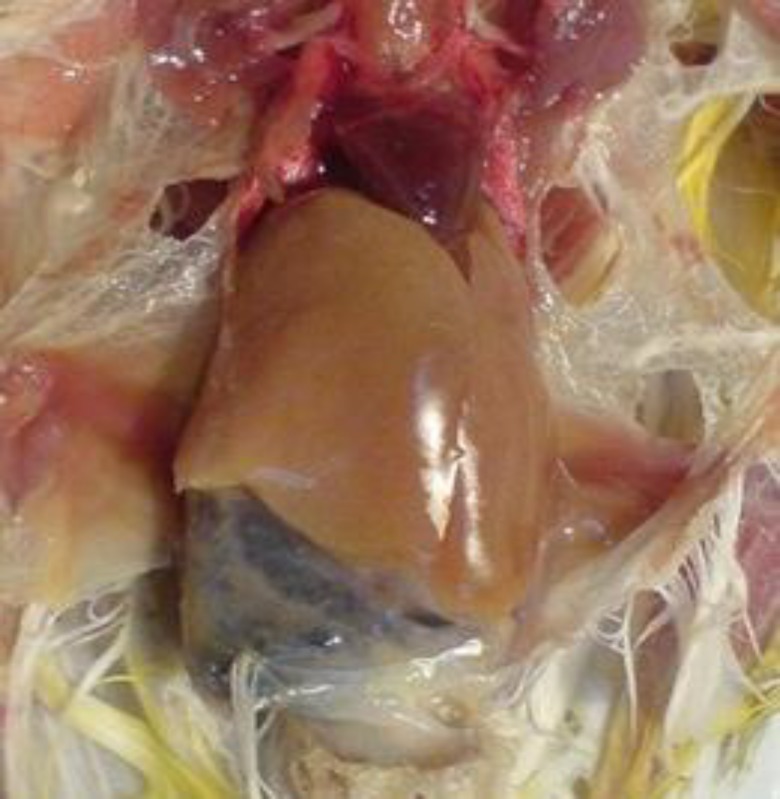
Enlarged yellowish brown pale liver in the affected canary. Note the loss of coronary adipose tissue and the dark mucoid contents in the intestines.

A dead female canary, found by its owner the night before presentation, was submitted for postmortem examination. At necropsy, the bird was extremely cachectic with poor body condition. Mild hyperkeratosis of the feet skin was obvious. Nearly no subcutaneous, abdominal, and/or coronary adipose tissue was seen. In alimentary tract, some mucoid fluid was seen at the mucosal surface of empty moderately dilated proventriculus. The gizzard was almost free of feed and filled with some insoluble grit. The koilin layer was typically loose and could be peeled easily. The intestines were filled with dark brown/black contents resembling antemortem hemorrhagic diathesis. The liver was significantly pale (yellow to light brown) and swollen (Fig. 1). Pale light pink/yellow spleen (about 10 mm) was observed. Both spleen and liver were fragile. The kidneys had pale creamy appearance. The ovary was inactive with follicles less than 1 mm.

**Fig. 2:**
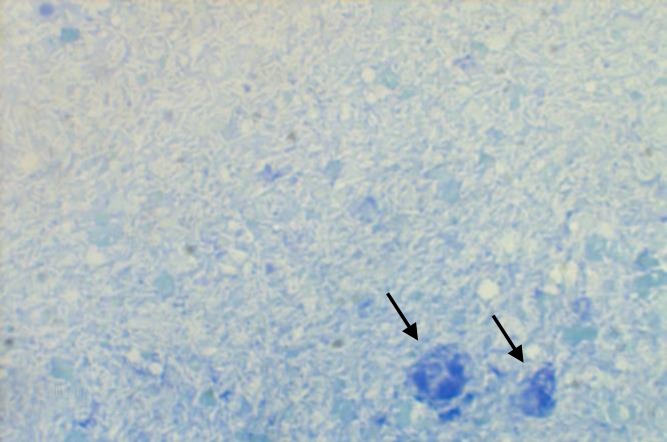
Liver contact smear. Degenerated cells (Black arrows). Note numerous unstained ghost-like bacilli covering all over the smear in the background, (Giemsa, 1000×).

**Fig. 3:**
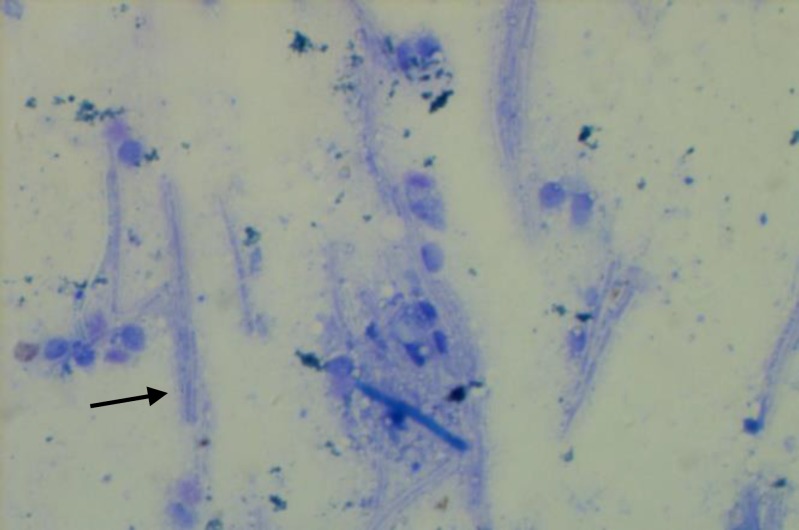
Scratch smear from proventriculus mucosa. Numerous huge bacilli (arrow) identical to *Macrorhabdus ornithogaster* can be seen, (Giemsa, 1000×).

**Fig. 4:**
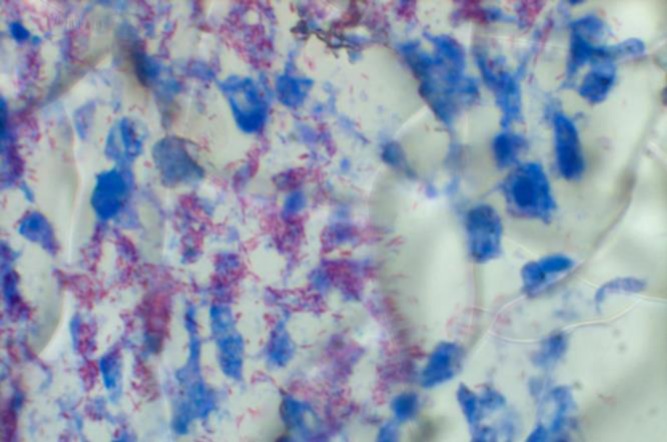
High magnification of thin histologic section of the liver. Too many tiny red acid fast bacilli scattered within the tissue, (Ziehl-Neelsen, 1000×).

In Giemsa stained contact smears of different organs, both liver and spleen had basophilic background. Severe massive necrosis and degeneration of hepatocytes and pyknotic hepatocyte nuclei were observed. Many small round nuclei without any nucleoli resembling lymphocytes were obvious. Some ghost-like unstained micro-organisms resembling acid-fast bacilli were seen in both cytoplasmic background and intracellularly in the liver (Fig. 2) and also in the spleen. In the lung many juvenile polychromatic erythrocytes were indicative of progressive regenerative anemia. In both wet smear and stained smear acquired from proventricular mucosa, many giant bacilli resembling *M. ornithogaster* were seen (Fig. 3). Numerous acid fast bacilli were histologically observed using Ziehl-Neelsen stained thin sections of liver (Fig. 4), spleen, lung and kidney. No bacteria could be isolated from liver and heart blood using MacConkey and blood agar media.

Histopathologic findings in the liver, revealed massive diffuse necrosis with diffuse infiltration of mononuclear inflammatory cells, mostly lymphocytes and plasma cells with some macrophages and epithelioid cells with diffusely scattered amorphous basophilic necrotic materials throughout the liver tissue. Spleen was obviously hypocellular with some mononuclear inflammatory cells. Within lung section, heavy infiltration of mononuclear cells, lymphoplasma cells, and some macrophages with some foci of anthracosis were obvious. In the kidney, severe massive necrosis with almost no normal tubular tissue was seen. The remaining cells appeared foamy. The proventriculus mucosa was lined with many huge bacilli (*M. ornithogaster*) and mild sub-mucosal mononuclear infiltration. In the gizzard a necrotic center was seen at the muscular layer with few giant cells and mononuclear cells surrounding it.

Based on the findings, atypical avian tuberculosis was diagnosed along with mycotic (yeast) gastritis due to *M. ornithogaster* infestation. As long as the affected bird had been isolated in an individual cage separate from other birds of the flock, the owner was recommended to clean and disinfect the cage and the entire enclosure thoroughly. 

To control the macrorhabdosis, 10 mL per L apple vinegar was administered via the drinking water for two weeks. No mortalities in the canaries have been reported for the next four months. The bird keepers were informed about the zoonotic potential of the infection. 

## Discussion

As emphasized in the literature, some species of *Mycobacteria* are the main cause of non-tuberculoid form of avian mycobacteriosis. In this article concurrent atypical avian tuberculosis and macrorhabdosis in a canary was diagnosed. The diagnosis was based on microscopic findings suggestive of avian mycobacteriosis and confirmed by Ziehl-Neelsen staining. In cytology of liver and spleen, some ghost-like unstained micro-organisms resembling *Mycobacterium sp.* were seen that were confirmed as an acid-fast bacteria using Ziehl*-*Neelsen staining method. 

Avian tuberculosis is an important bacterial disease in nearly all birds^13^^,^^17^ and some mammalian species,^18^ which has zoonotic potential for human.^12^.Normal appetite, contrary to poor body condition is the most common sign that is seen in infected birds. A presumptive diagnosis of the disease could be made based on the gross lesions. Tubercules or granulomas are good indicator of mycobacterial diseases but because of different pathologic changes, from typical to atypical lesions,^13^^,^^14^ cytology using different staining methods are needed to confirm or rule out tuberculosis. Several reports indicated that nonspecific postmortem picture, with an enlarged liver and lack of visible granulomatous lesion could be characteristic of mycobacteriosis in pet birds.^13^ Hence demonstration of acid-fast bacilli in smears or histologic sections of grossly altered organs confirms the diagnosis in such cases,^2^^,^^15^ In many cases of exotic pet birds,^4^^,^^6^^,^^19^ tuberculosis is found incidentally during post mortem examination.^3^ Canaries were first bred in captivity in the 17^th^ century. They are popular pet bird because of their distinct singing capabilities, beautiful and colorful plumage and their remarkable ability to cope with captive situation. Different species of *Mycobacterium* like *M. avium*,^20^ *M. genavense*,^21^^,^^22^* M. Tuberclosis,*^10^ *M. bovis*^23^ have public health significance and could be responsible for tuberculosis in human especially in very young, geriatric or immunosuppressed persons.^12^ Manarolla* et al*. reported atypical avian mycobacteriosis in eight canaries during a 20 years survey, all of them caused by *M. genavense* and had non- tuberculoid lesions.^6^ Hoop reported classical form of avian tuberculosis in a canary caused by *M. tuberculosis*.^11^ Ramis *et al*. described *M. genavense* infection in two canaries, from a collection of 50 adult canaries.^4^ Same as our case, affected birds had non-specific clinical signs and gross lesions and attempts to cultivate the agent were un-successful. They detected *M. genavense* DNA using PCR. Portaels *et al*. during 11 years survey in birds kept in a zoo, confirmed avian tuberclosis caused by *M. genavense* in 27 birds from different species.^3^ Most of them belonged to Passeriformes (12 cases). They concluded that *M. genavense* should be suspected in those birds died suddenly without previous symptoms, with non-specific gross findings and fastidious acid fast bacilli in tissues.

*Mycobacterium paratuberculosis, *the causal agent of Johne’s disease, and *M. bovis *the causative agent of bovine tuberculosis are some of the etiologies of avian tuberculosis.^12^ For exact identification and characterization of the causative *Mycobacterium* sp., different isolation methods and molecular techniques in adequate biohazard containment are needed.^24^ In the presented case no additional tests were performed to identify and characterize the etiologic *Mycobacterium *species, but according to the abundant numbers of acid-fast bacilli within the affected tissue^13^ and histopathologic nature of the affected tissues that almost exclusively *M. genavense *is associated with the non-tuberculoid form of avian mycobacteriosis,^6^^,^^14^ this organism is more probable. It may be suggested to avian veterinarians and pathologists to always prepare simple, feasible and low cost cytologic contact smears from different organs particularly liver, spleen, and lung. As it was shown in this case, these smears could simply help clinicians to find the definitive etiology of the condition.

Macrorhabdosis, formerly known as megabacteriosis, is a mycotic infection of gastrointestinal tract of many avian species which is caused by *M. ornithogaster*.^25^ It is often a chronic and wasting disease which might concurrently occur along with other infectious and non-infectious diseases. As its isolation is difficult and mostly unsuccessful,^26^ the diagnosis is commonly based on microscopic investigation and demonstration of large number of yeasts in the proventricular-ventricular junction.^25^ Macrorhabdosis has been previously reported in budgerigars (*Melopsittacus undulatus*) in Iran.^27^^,^^28^ Most *M. ornithogaster* infections cause little detectable disease however birds with megabacteriosis appear to be susceptible to other diseases such as combined megabacteriosis and trichomoniasis in budgerigars, heavy feather mite infestation, severe cnemidocoptic mange (scaly face), and chlamydiosis.^26^ The role of *Megabacteria* in depressing the immune system and allowing secondary infections is still unclear.

In many countries there are long-established legislation and instructions regarding the surveillance and monitoring of mycobacteriosis in cattle herds. In order to control these organisms in all animal populations, pet birds should be considered as a potential source of the infection. On the other hand, veterinarians should be aware of possibility of the occurrence of the atypical form of the disease in different bird species especially pet passerines and the appropriate approaches should be taken to diagnose these infections. 
